# Case Report: A combination of chimeric *CYP11B2/CYP11B1* and a novel p.Val68Gly *CYP11B*1 variant causing 11β-Hydroxylase deficiency in a Chinese patient

**DOI:** 10.3389/fendo.2023.1216767

**Published:** 2023-11-13

**Authors:** Jialin Li, Fenglan Zhang, Miao Xu, Hao Qiu, Cheng Zhou, Li Li, Lan Qin

**Affiliations:** ^1^Department of Endocrinology and Metabolism, The First Affiliated Hospital of Ningbo University, Ningbo, China; ^2^Clincal Genomics Center, Dian Diagnostics Group Co., Ltd., Hangzhou, China; ^3^Department of Urology, The First Affiliated Hospital of Ningbo University, Ningbo, China

**Keywords:** 11β-hydroxylase deficiency, molecular diagnosis, whole exome sequencing, chimeric CYP11B2/CYP11B1, missense variant

## Abstract

**Introduction:**

11β-Hydroxylase deficiency (11β-OHD, OMIM#202010) is the second most common form of congenital adrenal hyperplasia (CAH) caused by pathogenic variants in the *CYP11B1* gene. Both single nucleotide variations (SNV)/small insertion and deletion and genomic rearrangements of *CYP11B1* are important causes of 11β-OHD. Among these variant types, pathogenic *CYP11B2*/*CYP11B1* chimeras only contribute to a minority of cases. Heterozygote cases (chimera combined with SNV) are very rare, and genetic analysis of these cases can be challenging.

**Case presentation:**

We presented a suspected 11β-OHD female patient with incomplete virilization, adrenal hyperplasia, and hypokalemia hypertension. Whole exome sequencing (WES) revealed that the patient carried both a chimeric *CYP11B2/CYP11B1* and a novel missense variant, NM_000497.4: c.203T>G, p.Val68Gly (chr8:143961027) in *CYP11B1*, which were confirmed by CNVplex and Sanger sequencing, respectively. The patient’s manifestations and genetic findings confirmed the diagnosis of 11β-OHD, and oral dexamethasone was administered as a subsequent treatment.

**Conclusion:**

This report showed a rare *CYP11B2*/*CYP11B1* chimera combined with a novel missense variant in a 11β-OHD female patient. The result expands variant spectrum of *CYP11B1* and suggests that both chimera and *CYP11B1* variant screening should be performed simultaneously in suspected cases of 11β-OHD. To our knowledge, this is the first report about *CYP11B2*/*CYP11B1* chimera detected by WES analysis. WES combined with CNV analysis is an efficient method in the genetic diagnosis of this rare and complex disorder.

## 1 Introduction

Congenital adrenal hyperplasia (CAH) is an autosomal recessive disorder caused by a deficiency in enzymes required for the synthesis of cortisol from cholesterol (1). The most common form of CAH, accounting for 95% of cases, is 21-hydroxylase deficiency (21-OHD) (2). The second most common form of CAH is 11β-hydroxylase deficiency (11β-OHD), which accounts for approximately 5-8% of cases (3). 11β-hydroxylase converts 11-deoxycortisol and 11-deoxycorticosterone (DOC) to cortisol and corticosterone. Deficiencies in this enzyme lead to increased levels of 11-deoxycortisol and DOC, which are shunted into adrenal androgen synthesis pathways. The accumulation of DOC and testosterone causes hypertension and virilization in females or precocious puberty in males. Inadequate cortisol production stimulates the release of adrenocorticotrophic hormone (ACTH) as a compensatory mechanism, leading to subsequent adrenal hyperplasia (4, 5).

11β-hydroxylase and aldosterone synthase are encoded by the *CYP11B1*(OMIM#610613) and *CYP11B2* (OMIM#124080) genes, respectively, both of which consist of nine exons and share 95% exonic sequence homology and 90% intronic sequence homology. These genes lie tandemly arranged approximately 40 kb apart on chromosome 8q24 (6). To date, more than 200 pathogenic/likely pathogenic alterations of the *CYP11B1* gene associated with 11β-OHD have been reported in the ClinVar Database (https://www.ncbi.nlm.nih.gov/clinvar/). Most of the variants are missense, nonsense, frameshift, and splice variants. Moreover, the deletion of *CYP11B1* or chimeric *CYP11B2/CYP11B1* gene has been found in a few 11β-OHD patients (7).

With the extensive development of next-generation sequencing (NGS), whole exome sequencing (WES) has become the first-line diagnostic test in most monogenic disorders (8). Some algorithms have been designed to detect copy number variations (CNVs) based on the coverage depth of capture sequencing data, enabling the detection of CNVs larger than 200 kb. However, the reliability of these algorithms in detecting smaller CNVs is limited (9). The high degree of sequence similarity between *CYP11B1* and its homologous gene *CYP11B2* poses unique challenges for detecting small *CYP11B1* deletions or chimeric *CYP11B2*/*CYP11B1* through WES.

In this study, we reported a Chinese patient with classical manifestations of 11β-OHD resulting from compound heterozygous variants, including a novel missense variant NM_000497.4: c.203T>G, p.Val68Gly (chr8:143961027) in *CYP11B1* and a rare chimeric *CYP11B2*/*CYP11B1*. This study expands the variant spectrum of *CYP11B1* and demonstrates that a single WES test combined with WES based CNV analysis can be used effectively for the SNV/InDel identification and the chimeric *CYP11B2*/*CYP11B1* analysis.

## 2 Case presentation

**Figure 1:**
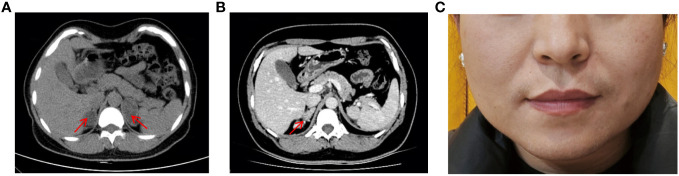
Masculine features and Abdominal CT scan of patient. **(A)** Abdominal CT scan showed bilateral adrenal hyperplasia, with a nodule on the right approximately 27×22mm and a nodule on the left approximately 42×28mm. **(B)** Abdominal CT scan revealed recurrence of the right adrenal gland hyperplasia two years after surgery. **(C)** Physical examination revealed greasy skin pigmentation, facial acne, and slight mustache on the upper lip.

The patient is a Chinese woman (46, XX) from a non-consanguineous family. She has one healthy younger brother. She was taller than her peers during childhood, but her growth did not accelerate during subsequent adolescence. She did not experience her first menstrual period until the age of 20. She underwent surgical treatment for “abnormal external genitalia” due to sexual dysfunction, but the specific diagnosis and surgical procedure are unknown. She has been unable to conceive since her marriage at the age of 26. In May 2019, she was admitted to the hospital with suspected bilateral adrenal tumors. Abdominal computed tomographic scan revealed bilateral adrenal multiple nodular hyperplasia (**Figure 1A**). After two surgeries, the left and right adrenal tumors were successfully removed. Partial adrenal was preserved on both sides to minimize the risk of adrenal insufficiency.

After the second surgery, she experienced irregular menstruation. Two and a half years later, she was admitted to the endocrinology department. Abdominal-enhanced CT scan showed structural disorder in the right adrenal gland area, with spotted and striped shadows (**Figure 1B**). Physical examination revealed greasy skin pigmentation, facial acne, and slight mustache on the upper lip (**Figure 1C**). Physical examination showed hypertension (145/100 mmHg), laboratory data showed decreased plasma potassium and aldosterone but elevated levels of adrenocorticotrophic hormone (ACTH), 11-deoxycorticosterone (DOC), 17-hydroxyprogesterone (17-OHP), androstenedione, Dehydroepiandrosterone (DHEA), and testosterone. The results of 1-day medium-dose dexamethasone androgen suppression test showed that 17-OHP, ACTH, androstenedione, DHEA, and testosterone were significantly suppressed (10) (**Table 1**). The external manifestations and biochemical indicators of patient were all suggestive of an 11β-OHD diagnosis.

**Table 1:** The laboratory test of the patient.

Laboratory examination	Patient value		Reference range
Potassium (mmol/L)	3.03		3.5-5.3
Cortisol (mmol/L)	247.63 (8am)		185.19-624.66
	114.44 (4pm)		
	21.44 (0am)		
ACTH (pmol/L)	72.59 (8am)		1.59-13.94
	18.16 (4pm)		
	3.04 (0am)		
Aldosterone (pg/ml)	<20		50-313
PRA (ng/ml/h)	0.42		0.25-5.82
DOC (pg/ml)	7810.2		≤180
Testosterone (pg/ml)	3409.8		80-600
Estradiol (pmol/L)	256		180-1068
Prolactin (mIU/L)	586.25		70.81-566.46
Progesterone (nmol/L)	9.31		16.41-59.02
LH (IU/L)	0.17		1.2-12.86
FSH (IU/L)	5.37		1.79-5.12
Laboratory examination	Pre-dexamethasone treatment	Post-dexamethasone treatment	Reference range
17-OHP (pg/ml)	11903.3	<100	<800
ACTH (pmol/L)	72.59	0.67	1.59-13.94
Androstenedione (pg/ml)	>10000	1417.2	300-2000
DHEA (pg/ml)	3612.3	982.2	<10000
DHEAS (pg/ml)	1.44×10^6	0.76×10^6	0.45-2.95×10^6
Testosterone (pg/ml)	3409.8	479.2	80-600

ACTH: Adrenocorticotrophic hormone. PRA: Plasma renin activity. DOC: 11-deoxycorticosterone. LH: Luteinizing hormone. FSH: Follicle-stimulating hormone.17-OHP: 17-hydroxyprogesterone. ACTH: Adrenocorticotrophic hormone, DHEA: Dehydroepiandrosterone. DHEAS: Dehydroepiandrosterone sulfate.

**Figure 2:**
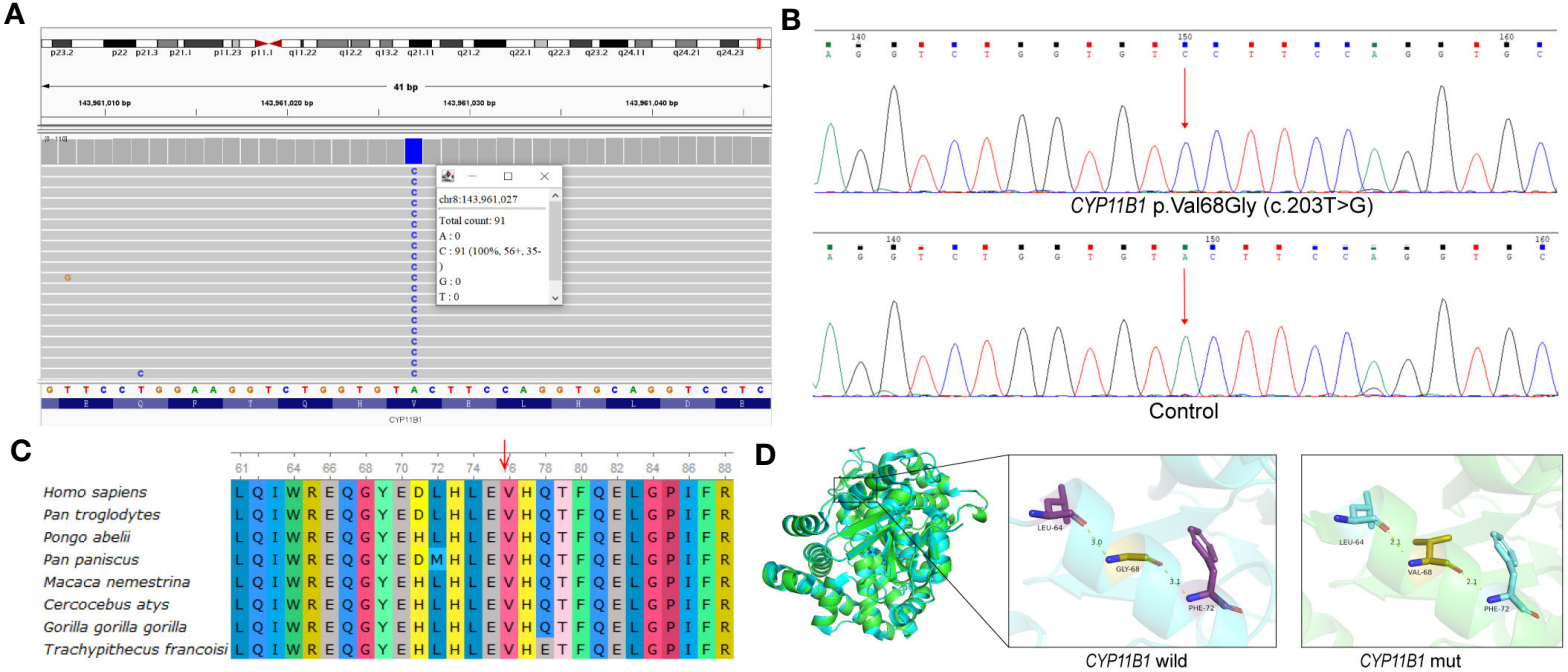
p.Val68Gly of the *CYP11B1* in patient. **(A)** Visualization of variant in *CYP11B1* using IGV. Apparently, the patient was a homozygous variant, c.203T>G, located in exon 1. **(B)** The sequencing chromatogram of the variant in *CYP11B1*. Arrow indicates mutant nucleotide (c.203T>G). **(C)** Conservation prediction of this mutant amino acid among different species. **(D)** Three-dimensional structure of wild-type *CYP11B1* and mutant-type *CYP11B1*. The yellow dotted line represents hydrogen bond.

**Figure 3:**
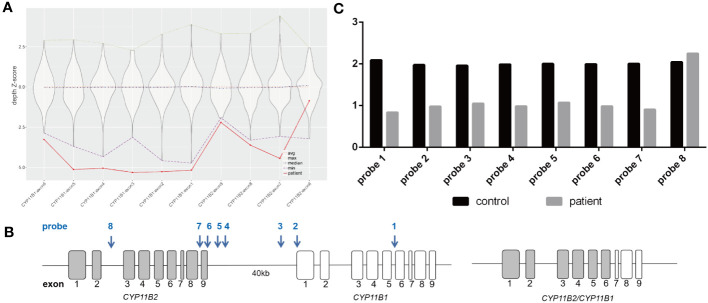
The chimeric *CYP11B2/CYP11B1* gene in patient. **(A)** The deletion covering exons 1-6 of *CYP11B1* and exons 7-9 in *CYP11B2* by whole exome sequencing. The violin plot illustrates the distribution of sequencing depth for normal reference samples on each exon, with the ordinate is the Z-score standardization for the sequencing depth. The dotted lines in different colors represent the maximum, average, median and minimum of sequencing depth Z-score in normal references. Solid line represents the sequencing depth Z-score of *CYP11B1* and *CYP11B2* in the proband sample. Specifically, the sequencing depth Z-score in exons 7-9 of *CYP11B2* and exon 1-6 of *CYP11B1* is lower than the minimum value in the normal references. **(B)** Schematic representation of the *CYP11B2* gene (exons displayed as grey boxes) and the *CYP11B1* gene (exons displayed as white boxes). In the investigated patient, the *CYP11B2*/*CYP11B1* chimera consisting of the promoter and exons 1–6 of *CYP11B2* and exons 7–9 of *CYP11B1*. Arrows indicate the position of the 8 specific probes of CNVplex.**(C)** Graphic report of CNVplex result in the patient and control. Deletion occurred in the position of seven probes, except for the position of the last probe (located in the intron 2 of *CYP11B2*).

To investigate the potential genetic pathogenic mechanism, whole exome sequencing was performed for the patient (**Supplementary Materials and Methods**). Initially, a novel homozygous missense variant in *CYP11B1*, NM_000497.4: c.203T>G (p.Val68Gly) was identified (**Figure 2A**). The newly identified missense variant was in exon 1. Then the variant was further confirmed by sanger sequencing (**Figure 2B**; **Supplementary Materials and Methods**). Furthermore, based on our WES-CNV analysis pipeline, a speculative *CYP11B1* and *CYP11B2* deletion was screened out (chr8:143957127-143994301). The deletion covers exon 1 to exon 6 of *CYP11B1* and exon 7 to exon 9 of *CYP11B2* (**Figure 3A**), resulting in the formation of a single hybrid gene consisting of the promoter and exons 1-6 of *CYP11B2* and exons 7-9 of *CYP11B1* (**Figure 3B**). Therefore, it’s reasonable to assume that p.Val68Gly of *CYP11B1* is a heterozygous variant rather than a homozygous variant.

The p.Val68Gly variant has not been recorded in several databases, including ClinVar (https://www.ncbi.nlm.nih.gov/clinvar/), Human Gene Mutation Database (HGMD, https://www.hgmd.cf.ac.uk/), PubMed (https://pubmed.ncbi.nlm.nih.gov/), and MasterMind (https://mastermind.genomenon.com/), indicating that this variant is novel. The Genome Aggregation Database (gnomAD, http://gnomad-sg.org/) does not include the frequency of this variant in normal East Asian populations, which indicates that the allele frequency of this variant is extremely low. To evaluate the pathogenicity of the newly identified variant, several prediction tools were used, in which the REVEL score was 0.339, and the ClinPred score was 0.3473. SIFT and Polyphen2 predicted the variant to be damaging and benign, respectively. Multiple alignments of *CYP11B1* suggest that the amino acidic residue, Val68, is conserved at this position across various species (**Figure 2C**). The prediction of protein three-dimensional structure revealed that Val68 residue locates in an α-helix, and p.Val68Gly causes a change in hydrogen bond length, which may lead to alterations in protein conformation and stability (**Figure 2D**). According to the ACMG guidelines, the classification for this variant was uncertain significance (PM2+PP3+PP4) (11). Due to the presence of a deletion-type allele, PM3 can be considered. Totally, it is recommended that the variant classification of NM_000497.4: c.203T>G (p.Val68Gly) in *CYP11B1* variant can be upgraded to likely pathogenic (PM2+PM3+PP3+PP4).

CNVplex was used to validate the deletion (exon 1 to exon 6 of *CYP11B1* and exon 7 to exon 9 in *CYP11B2*) (**Supplementary Materials and Methods**). The results showed significant copy number loss in 7 out of 8 groups of probes compared to the control group (**Supplementary Table 1**), while the other probe showed a normal copy number outside of the potential deletion region (**Figure 3C**). This result confirmed the reliability of this deletion recognized by WES-CNV analysis.

## 3 Discussion

11β-Hydroxylase deficiency is the second most common cause of congenital adrenal hyperplasia (CAH), accounting for 5–8% after the more prevalent 21-hydroxylase deficiency. The clinical phenotypes of patients with 11β-OHD are complex and nonspecific (12, 13). Patients who do not receive a molecular diagnosis or an appropriate hormonal evaluation may be misdiagnosed as 21-hydroxylase deficiency or other adrenal hyperplasia (14). To determine the CAH classification accurately, a WES analysis and a long-range PCR based *CYP21A2* sequencing were ordered simultaneously. However, the results of the long-range PCR for *CYP21A2* did not reveal any variants. Whereas a novel p.Val68Gly variant and a chimeric *CYP11B2/CYP11B1* were discovered on different alleles of *CYP11B1* by WES.

*CYP11B1* and *CYP11B2* encoded homologues, and have distinct functions in cortisol and aldosterone synthesis, respectively. *CYP11B2*/*CYP11B1* chimeric genes have been shown to arise from unequal crossing over of the *CYP11B2* and *CYP11B1* during meiosis. The activity deficiency or impaired activity of aldosterone synthase and 11β-hydroxylase resulting from these chimeric genes are important reasons for 11β-OHD (15). After reviewing previous reports on the chimeric *CYP11B2/CYP11B1* gene, we collected data on twelve patients with the chimera (**Supplementary Table 2**). Six of these patients harbored the chimeric *CYP11B2/CYP11B1* gene located in intron 6 of *CYP11B2*. Our patient carried the same pathogenic *CYP11B2/CYP11B1* chimera, suggesting that it may be a popular rearrangement event in 11β-OHD patients. Interestingly, the other allele of *CYP11B1* contained a new disease-causing variation, p.Val68Gly, in our patient. Although we were unable to obtain blood samples from the patient’s parents and brother for pedigree study, the presence of a deletion-type allele confirmed that the patient harbors a compound heterozygous variation. In the future, further functional analysis for this missense variant will be meaningful.

Previous studies on molecular genetic testing for *CYP11B1* variants mainly used *CYP11B1*-specific PCR with the aid of several key SNPs between *CYP11B1* and *CYP11B2 (* 16–18). However, identifying these previous hybrid genes was time-consuming and not feasible for all laboratories, such as southern blot. In 2015, Menabò S used homemade MLPA probes to identify a novel chimeric *CYP11B2/CYP11B1* gene in a 11β-OHD patient (19), but this MLPA method has not been widely adopted by genetic laboratories. The first report of a chimeric *CYP11B2/CYP11B1* detected by next-generation sequencing, in which 276 genes associated with adrenal diseases were captured, was published in 2022 (20). In this study, whole exome sequencing, a more general method, was used to detect *CYP11B1* variants and the chimera simultaneously, which, to our knowledge, is the first report.

In conclusion, a novel missense variant, p.Val68Gly, and a rare chimeric *CYP11B2/CYP11B1* gene were simultaneously detected by WES analysis in the suspected 11β-OHD patient, which is consistent with the clinical phenotype. These results indicate that WES is an effective molecular genetic test for detecting SNV/Indel and copy number variations. This study has expanded the variant spectrum of *CYP11B1*, contributing to early and accurate diagnosis and treatment of 11β-OHD patients, and ultimately promoting better genetic counseling. However, due to the rarity of chimeric variants, there is only one patient in our research, which indicates that further research and validation in larger patient cohorts is still needed in the future.

## Data availability statement

The original contributions presented in the study are included in the article/**Supplementary Material**, further inquiries can be directed to the corresponding author/s.

## Ethics statement

The studies involving humans were approved by the ethics committee of the First Affiliated Hospital of Ningbo University, China. The studies were conducted in accordance with the local legislation and institutional requirements. The participants provided their written informed consent to participate in this study. Written informed consent was obtained from the individual(s) for the publication of any potentially identifiable images or data included in this article.

## Author contributions

JL and FZ designed the study. JL, MX, CZ, and LL diagnosed the patient, provided follow-up, and acquired clinical data. FZ, HQ, JL, and LQ wrote and revised the manuscript. All authors contributed to the article and approved the submitted version.
