# Supplementary material for: Case Report: A combination of chimeric CYP11B2/CYP11B1 and a novel p.Val68Gly CYP11B1 variant causing 11β-Hydroxylase deficiency in a Chinese patient
Source: Front Endocrinol (Lausanne). 2023 Nov 13;14:1216767. doi: 10.3389/fendo.2023.1216767 (PMC10679387; doi:10.3389/fendo.2023.1216767)
Supplement: Supplementary file 1 [file DataSheet_1.docx]

Supplementary Material

Case Report: A Combination of Chimeric *CYP11B2/CYP11B1* and a Novel p.Val68Gly *CYP11B*1 Variant Causing 11β-Hydroxylase Deficiency in a Chinese Patient

# Supplementary Materials and methods

## Whole exome sequencing and data analysis

Genomic DNA (gDNA) was extracted from the peripheral blood of patient using the QIAamp DNA Blood Midi KIT (Qiagen, Germany) following standard protocols. Whole exome sequencing of the patient was performed by DIAN Diagnostics Corporation (Hangzhou, China) using the IDT xGen Exome Research Panel v1.0 (Integrated DNA Technologies, America) with a paired-end read length of 150 bp. Sequencing was performed using a NovaSeq 6000 system (Illumina, America), with an average sequencing depth of 100× and an average coverage of 99.59%. The sequences were aligned to the human reference genome hg19, and variants were called using the Genome Analysis Toolkit and annotated using VEP and Snpeff. Sanger sequencing was employed to confirm the identified variants. The data interpretation followed the guidelines of the American College of Medical Genetics and Genomics (ACMG), which classified the variants into five categories: benign, likely benign, unknown significance, likely pathogenic, and pathogenic(1).

Copy number variations (CNVs) of WES (WES-CNV)were identified using CNVkit(2) and exon Hidden Markov model (XHMM)(3). The reference database for CNV identification was established with CNV negative samples detected by CNVseq. Sequence coverage and examination of split reads were visualized in Interactive Genome Viewer (IGV)(4).

## Sanger sequencing

gDNA of the patient was amplified and sequenced with specific primer pairs, as follows: Primer1 Forward (*CYP11B1* c.203T>G) 5’-CACTCCTTCCCCATCTTCCA-3’ and Primer1 reverse (*CYP11B1* c.203T>G) 5’-TTCTCCCATGACGTGATCCC-3’. An ABI 3500Dx automated sequencer (Applied Biosystems, California) was used to analyze the PCR products.

## CNVplex

CNVplex is a method based on the Multiplex Ligation-dependent Probe Amplification (MLPA) technique(5). In the CNVplex array, a total of 8 sets of probes were designed (**Supplementary Table 1, Figure 3C**). Each probe consists of a hybridization oligonucleotide that is complementary to the target and a universal PCR primer sequence to allow PCR amplification with fluorescent primer pairs. The amplification products were separated by fluorescence capillary electrophoresis, and the peak heights of each site were obtained by electrophoretic maps. The peak heights of samples are calculated and subsequently normalized to reference segments. Reagents for the denaturation, ligation, and subsequent PCR amplification were purchased from Genesky Diagnostics (Suzhou, China) and Takara (Dalian, China). Capillary electrophoresis was conducted with an ABI 3130XL genetic analyzer (Applied Biosystems Inc., Foster City, CA, USA), and the raw data were analyzed by GeneMapper 4.1 (Applied Biosystems). In CNVplex, peak ratios between 0.75 and 1.25 are considered normal. Ratios above 1.25 indicate the presence of copy number gain of the target sequence, while ratios below 0.75 indicate copy number loss of the target sequence.

# Supplementary Tables

**Supplementary Table 1: CNVplex probes specific for *CYP11B1* and *CYP11B2* genes.**

| **Probe name** | **chrom** | **start** | **end** | **seq5’** | **seq3’** |
| --- | --- | --- | --- | --- | --- |
| Probe 1 | 8 | 143957406 | 143957449 | ACGACAGAGCCCAAGACTTCAA | ATCCTAATGCCCATCCAAACCC |
| Probe 2 | 8 | 143961240 | 143961283 | GGCACCAGGCAAGATAAAAGGA | TTGCAGCTGAACAGGGTGGAGG |
| Probe 3 | 8 | 143961337 | 143961378 | AGACCGAGGGCAGAGGTCAG | GGCTGGAGGCAGGGACTCATTG |
| Probe 4 | 8 | 143992057 | 143992101 | CAAGGAAGCCATCTCTGAGGTCT | GTGCACCTTGTTGCCCCCTTAT |
| Probe 5 | 8 | 143993102 | 143993148 | CCCCTTCTCCAGCAAGCAGTGC | CCTCTGGACAGCTTGACTCTACTCC |
| Probe 6 | 8 | 143993513 | 143993555 | GGACAGAGGCTGGGTTTCCATC | TGGCCTGGTCAGTAGCCCATG |
| Probe 7 | 8 | 143993827 | 143993869 | GTGTGATTGACACCTGGGAACA | GTGGATGGGGCCTTGGTTGGT |
| Probe 8 | 8 | 143997741 | 143997782 | ACAGTGGTGACAGCCCCCAC | TCCATGGTAGGAAGGGAACGCT |

**Supplementary Table 2: Clinical and genetic characteristics of patients with the chimeric *CYP11B2*/*CYP11B1* gene.**

| Patient | Gender | Clinical characteristics | Variant 1 | Variant 2 | Heterozygosity | Reference |
| --- | --- | --- | --- | --- | --- | --- |
| 1 | F | Incomplete virilization, adrenal hyperplasia, and hypokalemic hypertension | Deletion of exons 7-9 of *CYP11B2* and exons 1-6 of *CYP11B1* | p.Val68Gly | Compound heterozygosity | This report |
| 2 | M | Penile enlargement, increased growth velocity, and elevated blood pressure | Deletion of exons 7-9 of *CYP11B2* and exons 1-6 of *CYP11B1* | - | Homozygosity | Portrat S.et al. 2001(6) |
| 3 | M | Sexual precocity, hypertensive, and hyperpigmentation of the skin | Deletion of exons 5-9 of *CYP11B2* and exons 1-4 of *CYP11B1* | IVS3+16G>T | Compound heterozygosity | Hampf M.et al 2001(7) |
| 4 | M | Acceleration of growth, precocious pubarche, and macrogenitalia | Deletion of exons 5-9 of *CYP11B2* and exons 1-4 of *CYP11B1* | - | Homozygosity | Ezquieta B.et al. 2004(8) |
| 5 | M | The development of pubic hair and accelerated growth | Deletion of exons 4-9 of *CYP11B2* and exons 1-3 of *CYP11B1* | p.Gly314Arg | Compound heterozygosity | Kuribayashi I.et al.2005(9) |
| 6 | M | Suspected testicular tumor, short stature, acne, and a mild hypertension | Deletion of exons 7-9 of *CYP11B2* and exons 1-6 of *CYP11B1* | - | Homozygosity | Xu L.et al.  2015(10) |
| 7 | M | Hypertension, bilateral adrenal nodular thickening, decrease in sperm count and motility | Deletion of exons 7-9 of *CYP11B2* and exons 1-6 of *CYP11B1* | - | Homozygosity | Xu L.et al.  2015(10) |
| 8 | M | Dark skin pigmentation, penile enlargement, and pubic hair development, hypertension | Deletion of exons 7-9 of *CYP11B2* and exons 1-6 of *CYP11B1* | p.Trp56Ter | Compound heterozygosity | Xu L.et al.  2015(10) |
| 9 | F | Ambiguous external genitalia, hypertension | Deletion of exons 3-9 of *CYP11B2* and exons 1-2 of *CYP11B1* | p.Ala306Val | Compound heterozygosity | Menabò S.et al.  2015(11) |
| 10 | M | Precocious pseudopuberty and hypokalemia hypertension | Deletion of exons 7-9 of *CYP11B2* and exons 1-6 of *CYP11B1* | p.Leu340Pro | Compound heterozygosity | Duan L.et al.  2018(12) |
| 11 | M | Hypertension, excessive skin darkness, acne, accelerated growth velocity and precocious puberty | Deletion of exons 7-9 of *CYP11B2* and exons 1-6 of *CYP11B1* | p.Arg454His | Compound heterozygosity | Xie H.et al.  2022(13) |
| 12, 13 | M, F | Pseudoprecocious puberty and hypokalemia hypertension | Deletion of part of exon 6 to exon 9 of *CYP11B2* and exon 1 to part of exon 7 of *CYP11B1* |  | Homozygosity | Xiong Y.et al.  2023(14) |

F: female. M: male

# Supplementary references

1. Richards S, Aziz N, Bale S, Bick D, Das S, Gastier-Foster J, et al. Standards and Guidelines for the Interpretation of Sequence Variants: A Joint Consensus Recommendation of the American College of Medical Genetics and Genomics and the Association for Molecular Pathology. Genet Med. 2015 May;17(5):405–24.

2. Talevich E, Shain AH, Botton T, Bastian BC. CNVkit: Genome-Wide Copy Number Detection and Visualization from Targeted DNA Sequencing. PLoS Comput Biol. 2016 Apr;12(4):e1004873.

3. Fromer M, Moran JL, Chambert K, Banks E, Bergen SE, Ruderfer DM, et al. Discovery and statistical genotyping of copy-number variation from whole-exome sequencing depth. Am J Hum Genet. 2012 Oct 5;91(4):597–607.

4. Thorvaldsdóttir H, Robinson JT, Mesirov JP. Integrative Genomics Viewer (IGV): high-performance genomics data visualization and exploration. Brief Bioinform. 2013 Mar;14(2):178–92.

5. Zhang X, Xu Y, Liu D, Geng J, Chen S, Jiang Z, et al. A modified multiplex ligation-dependent probe amplification method for the detection of 22q11.2 copy number variations in patients with congenital heart disease. BMC Genomics. 2015 May 8;16(1):364.

6. Portrat S, Mulatero P, Curnow KM, Chaussain JL, Morel Y, Pascoe L. Deletion Hybrid Genes, due to Unequal Crossing Over between CYP11B1 (11β-Hydroxylase) and CYP11B2(Aldosterone Synthase) Cause Steroid 11β-Hydroxylase Deficiency and Congenital Adrenal Hyperplasia1. The Journal of Clinical Endocrinology & Metabolism. 2001 Jul 1;86(7):3197–201.

7. Hampf M, Dao NT, Hoan NT, Bernhardt R. Unequal crossing-over between aldosterone synthase and 11beta-hydroxylase genes causes congenital adrenal hyperplasia. J Clin Endocrinol Metab. 2001 Sep;86(9):4445–52.

8. Ezquieta B, Luzuriaga C. Neonatal salt-wasting and 11 β-hydroxylase deficiency in a child carrying a homozygous deletion hybrid CYP11B2 (aldosterone synthase)–CYP11B1 (11 β-hydroxylase). Clinical Genetics. 2004;66(3):229–35.

9. Kuribayashi I, Nomoto S, Massa G, Oostdijk W, Wit JM, Wolffenbuttel BHR, et al. Steroid 11-beta-hydroxylase deficiency caused by compound heterozygosity for a novel mutation, p.G314R, in one CYP11B1 allele, and a chimeric CYP11B2/CYP11B1 in the other allele. Horm Res. 2005;63(6):284–93.

10. Xu L, Xia W, Wu X, Wang X, Zhao L, Nie M. Chimeric CYP11B2/CYP11B1 causing 11β-hydroxylase deficiency in Chinese patients with congenital adrenal hyperplasia. Steroids. 2015 Sep;101:51–5.

11. Menabò S, Boccassini S, Gambineri A, Balsamo A, Pasquali R, Prontera O, et al. Improving the diagnosis of 11β-hydroxylase deficiency using home-made MLPA probes: identification of a novel chimeric CYP11B2/CYP11B1 gene in a Sicilian patient. J Endocrinol Invest. 2016 Mar;39(3):291–5.

12. Duan L, Shen R, Song L, Liao Y, Zheng H. A novel chimeric CYP11B2/CYP11B1 combined with a new p.L340P CYP11B1 mutation in a patient with 11OHD: case report. BMC Endocr Disord. 2018 Apr 27;18:23.

13. Xie H, Yin H, Ye X, Liu Y, Liu N, Zhang Y, et al. Detection of Small CYP11B1 Deletions and One Founder Chimeric CYP11B2/CYP11B1 Gene in 11β-Hydroxylase Deficiency. Front Endocrinol (Lausanne). 2022 May 24;13:882863.

14. Xiong Y, Zeng Z, Liang T, Yang P, Lu Q, Yang J, et al. Unequal crossing over between CYP11B2 and CYP11B1 causes 11 β -hydroxylase deficiency in a consanguineous family. J Steroid Biochem Mol Biol. 2023 Oct;233:106375.
